# Point Mutations in Centromeric Histone Induce Post-zygotic Incompatibility and Uniparental Inheritance

**DOI:** 10.1371/journal.pgen.1005494

**Published:** 2015-09-09

**Authors:** Sundaram Kuppu, Ek Han Tan, Hanh Nguyen, Andrea Rodgers, Luca Comai, Simon W. L. Chan, Anne B. Britt

**Affiliations:** 1 Department of Plant Biology, University of California Davis, Davis, California, United States of America; 2 Plant Biology and Genome Center, University of California Davis, Davis, California, United States of America; Harvard University, UNITED STATES

## Abstract

The centromeric histone 3 variant (CENH3, aka CENP-A) is essential for the segregation of sister chromatids during mitosis and meiosis. To better define CENH3 functional constraints, we complemented a null allele in *Arabidopsis* with a variety of mutant alleles, each inducing a single amino acid change in conserved residues of the histone fold domain. Many of these transgenic missense lines displayed wild-type growth and fertility on self-pollination, but exhibited frequent post-zygotic death and uniparental inheritance when crossed with wild-type plants. The failure of centromeres marked by these missense mutation in the histone fold domain of CENH3 reproduces the genome elimination syndromes described with chimeric CENH3 and CENH3 from diverged species. Additionally, evidence that a single point mutation is sufficient to generate a haploid inducer provide a simple one-step method for the identification of non-transgenic haploid inducers in existing mutagenized collections of crop species. As proof of the extreme simplicity of this approach to create haploid-inducing lines, we performed an *in silico* search for previously identified point mutations in CENH3 and identified an *Arabidopsis* line carrying the A86V substitution within the histone fold domain. This A87V non-transgenic line, while fully fertile on self-pollination, produced postzygotic death and uniparental haploids when crossed to wild type.

## Author Summary

The centromeric histone protein, CENH3, plays an important role in chromosome segregation during mitosis and meiosis. Here we show that single amino acid changes in CENH3, while producing no obvious effect on mitosis or meiosis, affect segregation postzygotically upon outcrossing to plants carrying wild-type centromeres. This results in uniparental inheritance among some progeny, and seed death in a larger fraction of progeny. Interestingly, changes competent to induce haploid in Arabidopsis existed in a TILLING population and in unrelated plant species. Our findings have two major consequences. First, uniparental inheritance facilitates the production of haploid plants that can easily be doubled to produce completely homozygous lines in a single generation. Secondly, our findings suggest that natural variation in CENH3 may result in partial reproductive isolation, because chromosomes of the mutant parent from F1 hybrid progeny are culled during embryonic development, while no reproductive defects are observed in self-pollinated plants. We do not know if the same mutations are haploid-inducing in other species, but uniparental chromosome loss, and the seed abortion that accompanies it results in an outcrossing-specific penalty that could potentially be involved in reproductive isolation.

## Introduction

Accurate segregation of eukaryotic chromosomes into daughter cells requires the presence of a centromere. Centromeres are, in most species, a region on each chromosome that directs the assembly of the kinetochore during mitosis and meiosis. The kinetochore is a substantial molecular motor, consisting of hundreds of proteins, which regulates and drives the migration of sister chromatids (in mitosis) or homologous chromosomes (in meiosis I) to opposite poles of the cell [1–4]. Centromeres are largely thought to be determined epigenetically by the presence of nucleosomes containing the centromere-specific histone H3 variant CENH3 (aka, CENP-A) [5–9].

In stark contrast to conventional histones, which are among the most conserved proteins in eukaryotes, CENH3 is rapidly evolving [10]. CENH3 structure is divided into two domains, a highly variable (in length and sequence) N-terminal tail and the more conserved C-terminal Histone Fold Domain (HFD). Although a handful of amino acids are highly conserved at the N-terminus of the N-terminal tail domain, the rest of the tail is so rapidly evolving that it cannot be aligned even among fairly related clades. For example, among the eudicots known CENH3 N-terminal tails range in length from 23 to 194 amino acids [11]. The HFD is in contrast, relatively well conserved, although it displays signatures of adaptive evolution in some residues [10, 12]. Given the proven role of CENH3 in the specification of the centromere, it is of no surprise that null alleles, though transmissible, are lethal as homozygotes [13–16]. Similarly, defects in the localization of CENH3- either a failure to reload or promiscuous loading to more than one site per chromosome- would be expected to lead to severe genetic abnormalities. Defects in CENH3 loading have been shown to cause chromosomes instability in several organisms, including budding yeast, humans and *Arabidopsis* [17–19].

Manipulation of CENH3 itself also has dramatic effects on chromosome segregation, an outcome with both basic and applied significance [11, 20–22]. Swapping the CENH3 hypervariable N-terminal tail with that of histone H3.3-like and concurrent fusion to GFP (“*GFP*-tailswap”) produces in a partially sterile plants showing meiotic defects. Interestingly, when the *GFP*-tailswap line is crossed to the wild type, the chromosomes derived from the parent expressing this chimeric protein misseggregate during embryogenesis, resulting in elimination of the corresponding parental genome, producing haploid plants whose chromosomes were derived from only the wild-type CENH3 parent. Maheshwari et al [11], recently demonstrated that transgenic *CENH3* genes derived from progressively distant relatives (through the monocot *Z*. *mays*), can complement the lethality of a *cenh3* -/- null mutant of Arabidopsis, and the transgenic plants were fertile. However, when crossed with plants expressing wild-type *CENH3*, the progeny displayed various degrees of embryonic lethality, aneuploidy and haploidy. Missegregation affected only chromosomes from the parent expressing the distant CENH3.

Translation of these discoveries to haploid production in crops would accelerate trait mapping and plant breeding [23–25]. Implementing the *GFP*-tailswap or transgenic-complementation approach, however, requires two steps. First, a CENH3 knockout (KO) must be obtained, as the haploid induction trait conferred by the variant CENH3 is suppressed by the wild-type CENH3 protein. Second, this KO mutant must be complemented with the chimeric or trans-species transgene, a genetic modification likely to require expensive regulatory approval, which in some cases is unacceptable to the public.

These findings pose a basic question. Could a single amino acid change in CENH3 result in the missegregation syndrome, i.e. in a plant which is fertile on self-pollination, but whose centromeres malfunction when confronted zygotically with centromeres determined by wild-type CENH3? To address this, we decided to explore how single amino acid substitutions in CENH3 affect centromere function and chromosome segregation. Here, we show that changes in CENH3 sequence that could be derived naturally (or through simple chemical mutagenesis) can result in haploid induction upon hybridization, apparently without secondary effects on growth and fertility. This finding indicates that single amino acid changes at this rapidly evolving centromeric protein have dramatic consequences on the mutant ability to hybridize. At the same time, it provides a simple, non-transgenic tool for developing haploid inducers in crops.

## Results

**Fig 1:**
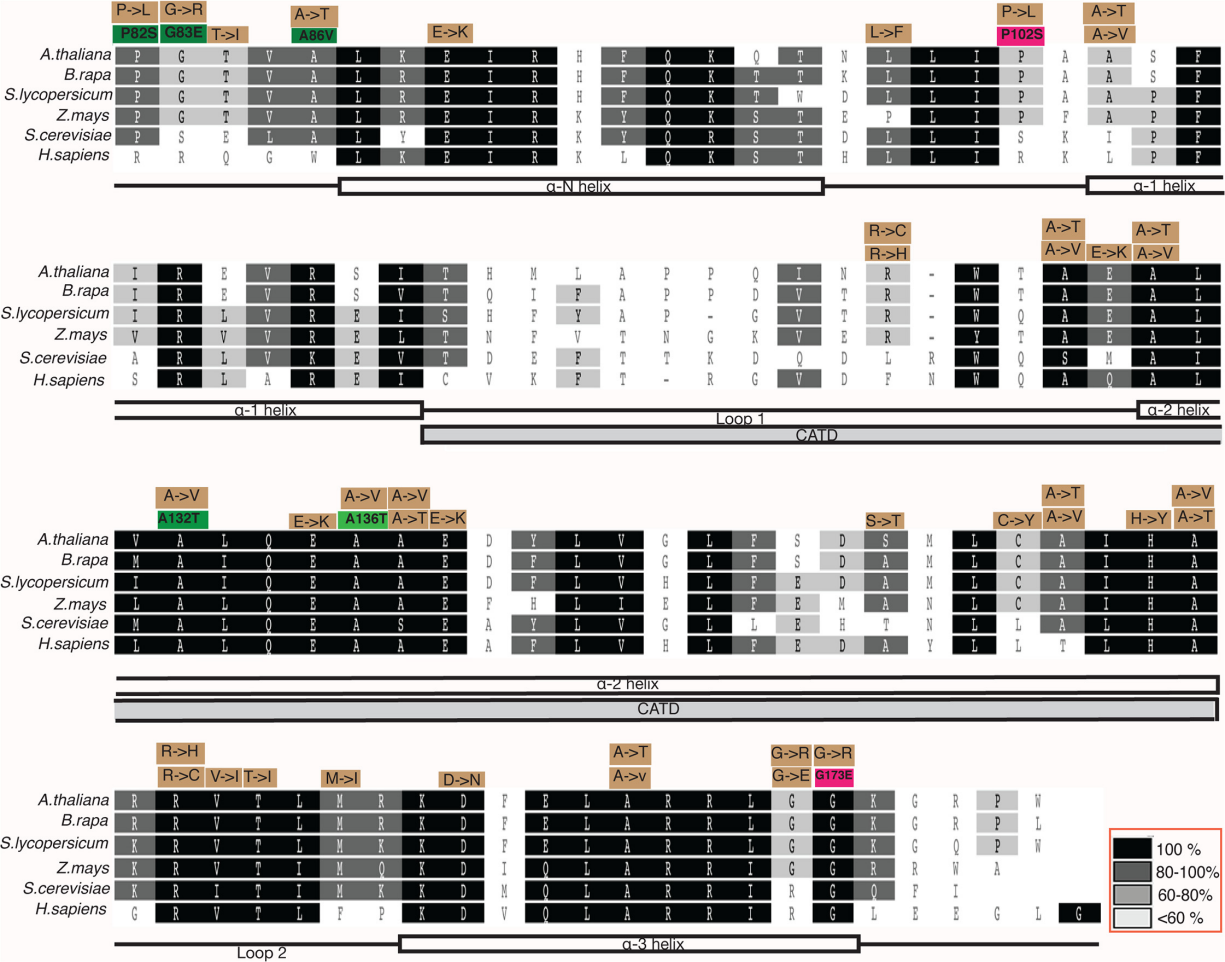
Multiple sequence alignment of CENH3 Histone Fold Domain (HFD) of *Arabidopsis thaliana*, *Brassica rapa*, *Solanum lycopersicum*, *Zea mays*, *Saccharomyces cerevisiae* and *Homo sapiens*. The annotations in the boxes above the alignment blocks indicate the single amino acid substitutions that can be mutated by G to A or C to T transition in four plant species (*A*. *thaliana*, *B*. *rapa*, *S*. *lycopersicum*, *Z*. *mays*). Green boxes indicate the point mutations that result in the induction of haploids and magenta boxes indicate point mutations that did not result in induction of haploids (at the scale measured here, Table 1) in *Arabidopsis thaliana*. The brown boxes are other EMS-inducible missense mutations identified in this study. Amino acid residue numbers within the green and magenta boxes correspond to positions of *Arabidopsis thaliana* CENH3. Scoring matrix: Blosum. Inset red box shows the similarity index.

*AtCENH3* consists of an N-terminal tail region and a C-terminal histone fold domain (HFD). To identify the conserved domains of CENH3 (and so identify particularly critical amino acids) we aligned the CENH3 protein sequences of over 50 plant species. The tail region is highly variable whereas the HFD is relatively conserved across species (S1 Fig), and for this reason we focused our attention on the HFD. We identified amino acids in *Arabidopsis thaliana*, as well as in cultivated dicot species *Brassica rapa*, *Solanum lycopersicum* and the monocot *Zea mays* that were conserved and could be mutated to produce the same amino acid change in all four species by G to A or C to T transition (reflecting the mutation spectrum of alkylating chemical mutagens). We identified 47 possible mutations in 30 amino acids in the HFD that fit these criteria (S1 Table). A comparison of CENH3s from these four plant species to CENH3s from yeast and human shows that some of these amino acids are conserved across kingdom (Fig 1).

To identify potentially important amino acid changes, we used SIFT [26, 27] to predict whether a substitution of one amino acid for another would be functionally tolerated. SIFT predicted that 38 of our candidates would not be tolerated while 9 were more benign (S2 Table). We selected six mutant alleles (Table 1) and tested their ability to transgenically complement a *cenh3-1* null mutation (the null allele is zygotic lethal), support fertility, and produce haploids upon crossing with wild-type *Arabidopsis*.

**Table 1:** Haploid induction and seed abortion frequency of transgenic and TILLING lines used in this study.

Line	Codon change	Amino acid change	Aborted seeds (%)	Haploids/Total progeny (%)
WT-HFD#1	No change	No change	0	0/199 (0)
WT-HFD#10	No change	No change	0	0/243 (0)
WT-HFD#15	No change	No change	0	0/163 (0)
M1#6	CCA→ TCA	P82S	15	8/334 (2.4)
M1#8	CCA→TCA	P82S	21	2/72 (2.7)
M1#11	CCA→TCA	P82S	20	11/435 (2.5)
M4#16	GGA→GAG	G83E	36	20/164 (12.2)
M4#18	GGA→GAG	G83E	28	18/197 (9.1)
M10#6	CCG→TCC	P102S	10	0/203 (0)
M10#19	CCG→TCC	P102S	0	0/115 (0)
M21#2	GCT→ ACG	A132T	4	3/475(0.63)
M21#2	GCT→ ACG	A132T	10	1/163(0.61)
M26#4	GCA→ACA	A136T	24	7/309 (2.26)
M47#15	GGA→GAA	G173E	0	0/207 (0)
**TILLING**	GCT →GTT	A86V	32	3/110 (2.72)
M7 # 3	GCT →GTT	A86V	33	9/232 (3.87)

Crosses using transgenic *cenh3-1* -/- plants carrying WT-HFD or *CENH3* point mutants (independently derived lines indicated by #) as well as a TILLING point mutant were assessed. The crosses featuring P82S, G83E, A132T, A136T, A86V (transgenic and TILLING) point mutations led to uniparental (maternal) genome elimination, producing paternal haploids. Lines derived from WT, P102S, and G173E did not produce haploids when crossed, at the scale investigated here (> 0.5% haploids).

**Fig 2:**
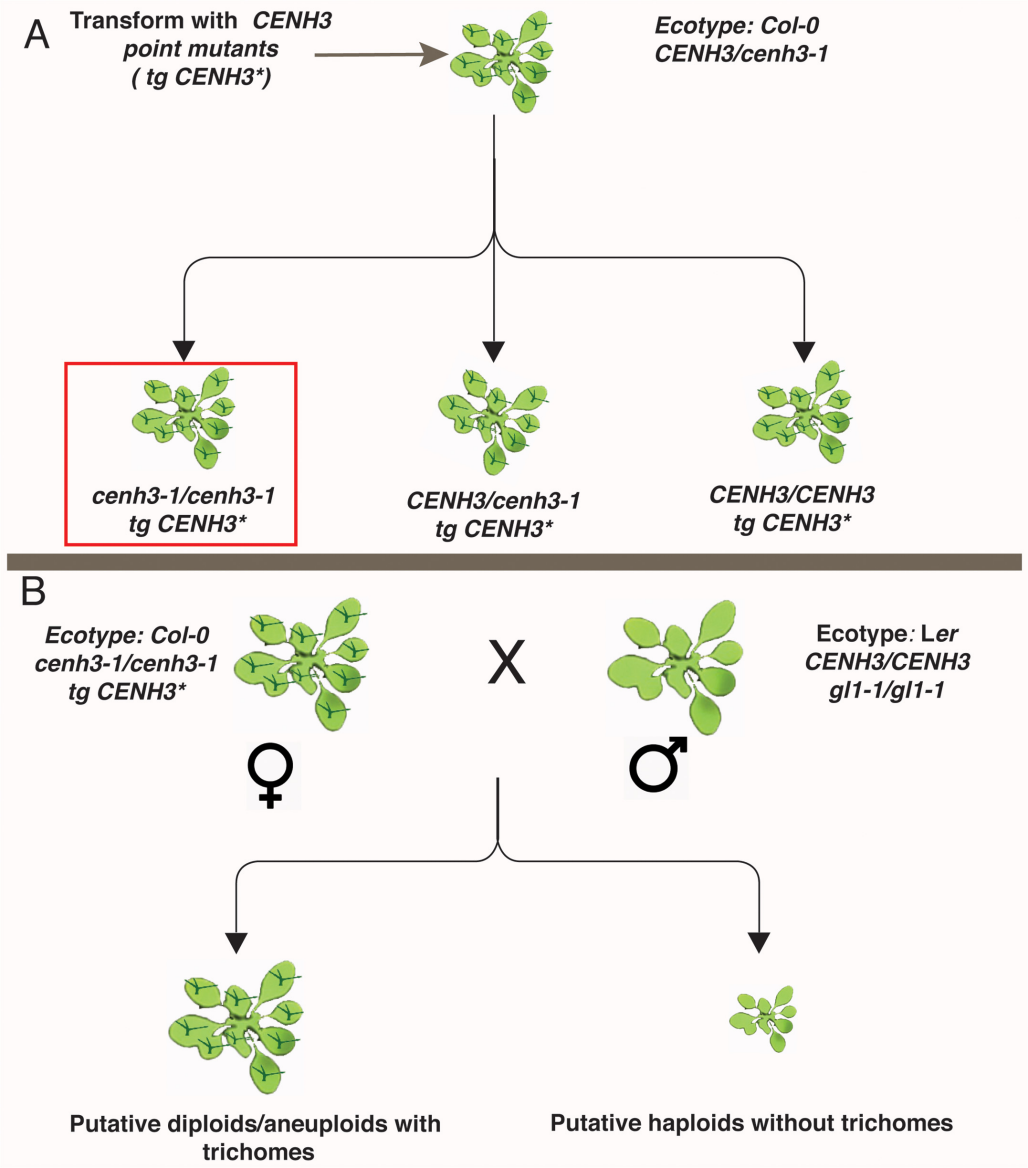
Schematic representation of transgenic *CENH3* point mutant transformation and crossing. (A) Steps involved in generation of transgenic *CENH3* point mutants in *cenh3-1/cenh3-1* background indicated in the red box. (B) *CENH3* point mutants in *cenh3-1/cenh3-1* background in Col-0 ecotype with trichomes (*GL1/GL1*) were used as female parent and crossed by *gl1-1/gl1-1* plants that carry wild-type allele for *CENH3* (*CENH3/CENH3*) in Landsberg *erecta* ecotype. The possible outcomes and their phenotype are represented below. Trichomes are represented in dark green. Uniparental paternal haploids do not have trichomes a feature that was used for identification of haploids. tg CENH3* stands for transgenic *CENH3* with point mutations.

In order to avoid lethality[16], our constructs were transformed into *CENH3*/*cenh3-1* plants and their offspring were screened for both the presence of the transgene and native *CENH3* genotype (Fig 2A and S2 Fig). To determine whether alteration in the level of expression of *CENH3* (caused by variable levels of expression of the transgene in independently derived transformants) leads to a haploid inducing effect, we generated a wild-type version of our transgene, using the same vector backbone. This transgene (*WT-HFD*) has the native *CENH3* promoter, native 5’ UTR and *CENH3* tail domain with a synthetic wild-type histone fold domain. Three independent insertion lines carrying WT-HFD were analyzed. In all three WT-HFD lines (*cenh3-1/cenh3-1* expressing *WT-HFD CENH3*) were able to complement the nullimorphic *cenh3-1* mutation without any obvious phenotypic effects. These *WT-HFD* plants were fully fertile, and produced 100% normal seeds upon self-pollination.

Transgenic plants expressing the single-amino acid substitutions P82S, G83E, P102S, A132T, A136T and G173E were also viable and fully fertile on self-pollination. These plants did not show any phenotypic difference compared to wild-type plants (S3 Fig). Analyses of pollen viability in these lines also showed that pollen from these transgenic point mutants appear normal (S4 Fig). Thus, the mutant transgenes were able to complement the *cenh3-1* mutation both mitotically and meiotically. To determine whether the complemented lines were haploid inducers, we crossed them by Landsberg *erecta glabrous1* (L*er gl1-1/gl1-1 CENH3/CENH3)* (Fig 2B). These recessive *er* (compact growth habit) and *gl1* (hairless leaves) mutations are on chromosome 2 and 3, respectively. Based on earlier research [21] we hypothesized that elimination of maternal chromosomes, might lead to the production of paternal haploids, which would then exhibit both the *erecta* and *glabrous* phenotypes. Crosses of our WT-HFD transgenics (*cenh3-1/cenh3-1* expressing *WT-HFD CENH3*) with tester line (L*er gl1-1/gl1-1 CENH3/CENH3)*, produced 100% normal seeds without obvious induction of seed death, a trait associated with haploid induction, and 100% of the F1 progeny displayed wild-type phenotype, indicating that they were diploids carrying both maternal and paternal chromosomes.

**Fig 3:**
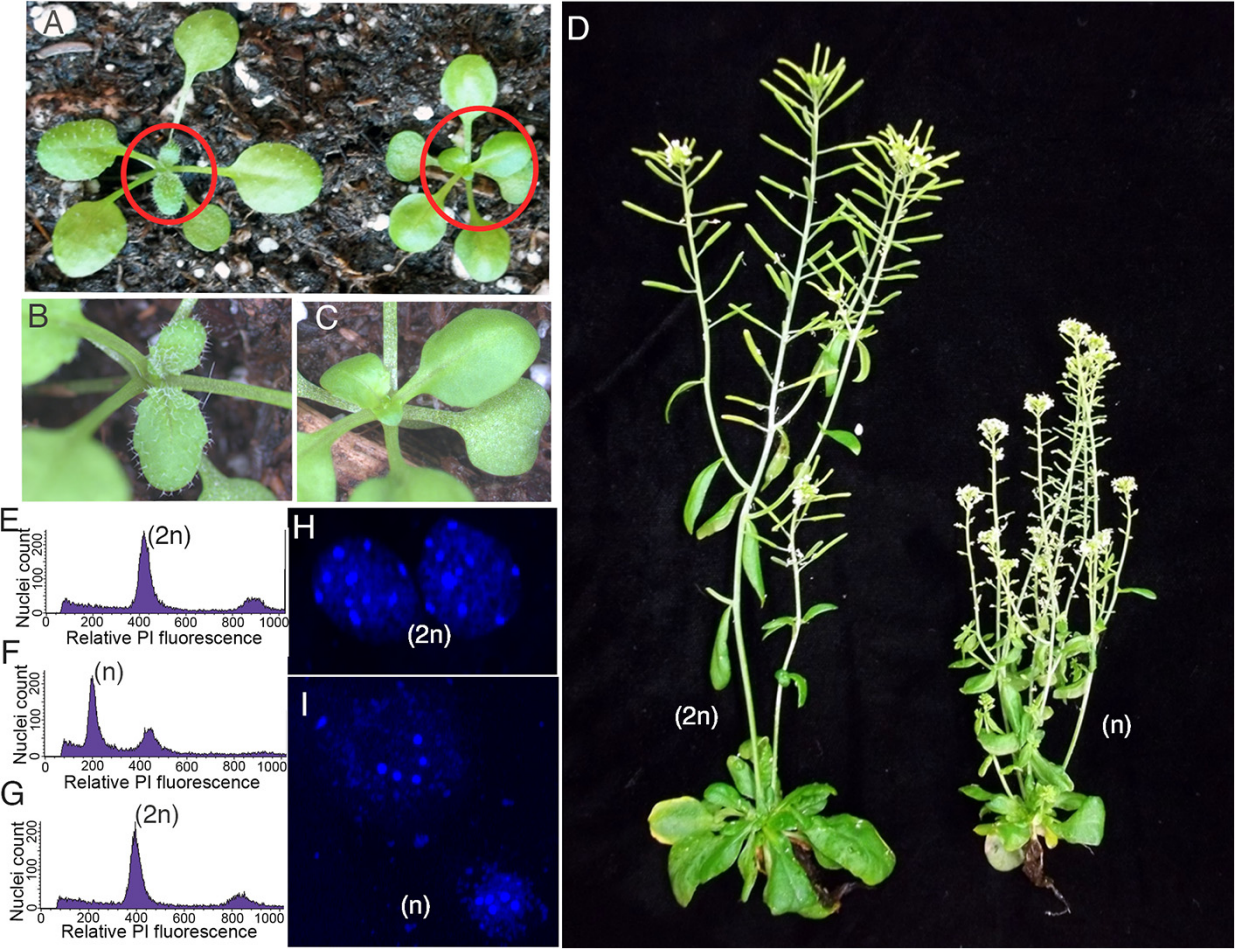
Haploid plants produced by genome elimination in crosses of *CENH3* point mutants by L*er gl1-1*. (A) Comparison of diploid hybrid with trichomes on the left and smaller haploid plant without trichomes on the right from haploid inducing cross. (B-C) Inset enlargements (region circled in red) show a diploid plant with trichomes on the left and a trichomeless haploid on the right. (D) Phenotype of a L*er gl1-1* haploid with undeveloped siliques on the right and a diploid L*er gl1-1* showing healthy siliques on the left. (E-G) Analysis of nuclei stained with propidium iodide (PI) by flow cytometry for a diploid control diploid (E), L*er gl1-1* glabrous haploid (F) offspring and a L*er gl1-1* doubled haploid (G). (H-I) DAPI stained nuclei of a diploid plant showing 10 chromocenters (H) and a haploid plant showing 5 chromocenters (I). Scale bars = 5 μm.

The mutant P82S lines (*cenh3-1/cenh3-1* expressing *P82S-CENH3)*, when crossed with the same tester pollen (L*er gl1-1/gl1-1 CENH3/CENH3)*, produced 15–20% dead seeds, and of the viable offspring 2–3% were both *erecta* and *glabrous*, consistent with loss of the dominant maternal markers. These putative haploid plants were smaller than corresponding diploids (Fig 3A), trichomeless (Fig 3B and 3C) and sterile (Fig 3D), also consistent with haploidy. Analysis of putative haploids from the point mutant line by flow cytometry against the diploid control confirmed their haploid status. A sample plot of diploid control and haploid from mutant P82S is shown (Fig 3E and 3F). Cytogenetic analyses confirmed haploid content, corresponding to 5 chromosomes *vs*. 10 in diploids (Fig 3H and 3I). Similarly, G83E (*cenh3-1/cenh3-1* expressing *G83E-CENH3)*, A132T (*cenh3-1/cenh3-1* expressing *A132T*-*CENH3)* and A136T (*cenh3-1/cenh3-1* expressing *A136T-CENH3*) point mutants, while somatically normal and fully fertile on self-pollination, produced both aborted seeds and flow cytometry-confirmed haploid progeny, on crossing with tester pollen (L*er gl1-1/gl1-1 CENH3/CENH3*) (Table 1). Notwithstanding the conservation of these amino acids among angiosperms (S2 Table) and the “not tolerated” prediction by SIFT, the phenotype of plants expressing the altered CENH3 in lieu of the wild-type CENH3 was indistinguishable from wild-type unless crossed with pollen carrying centromeres determined by wild-type CENH3. G173E (*cenh3-1/cenh3-1* expressing *G173E-CENH3*), another mutation predicted “not tolerated”, appeared to be wild-type even on crossing by wild-type pollen. Similarly, a 6^th^ mutation, P102S (*cenh 3-1/cenh3-1* expressing *P102S-CENH3*), was predicted to be tolerated and indeed displayed no effect on CENH3 function.

**Fig 4:**
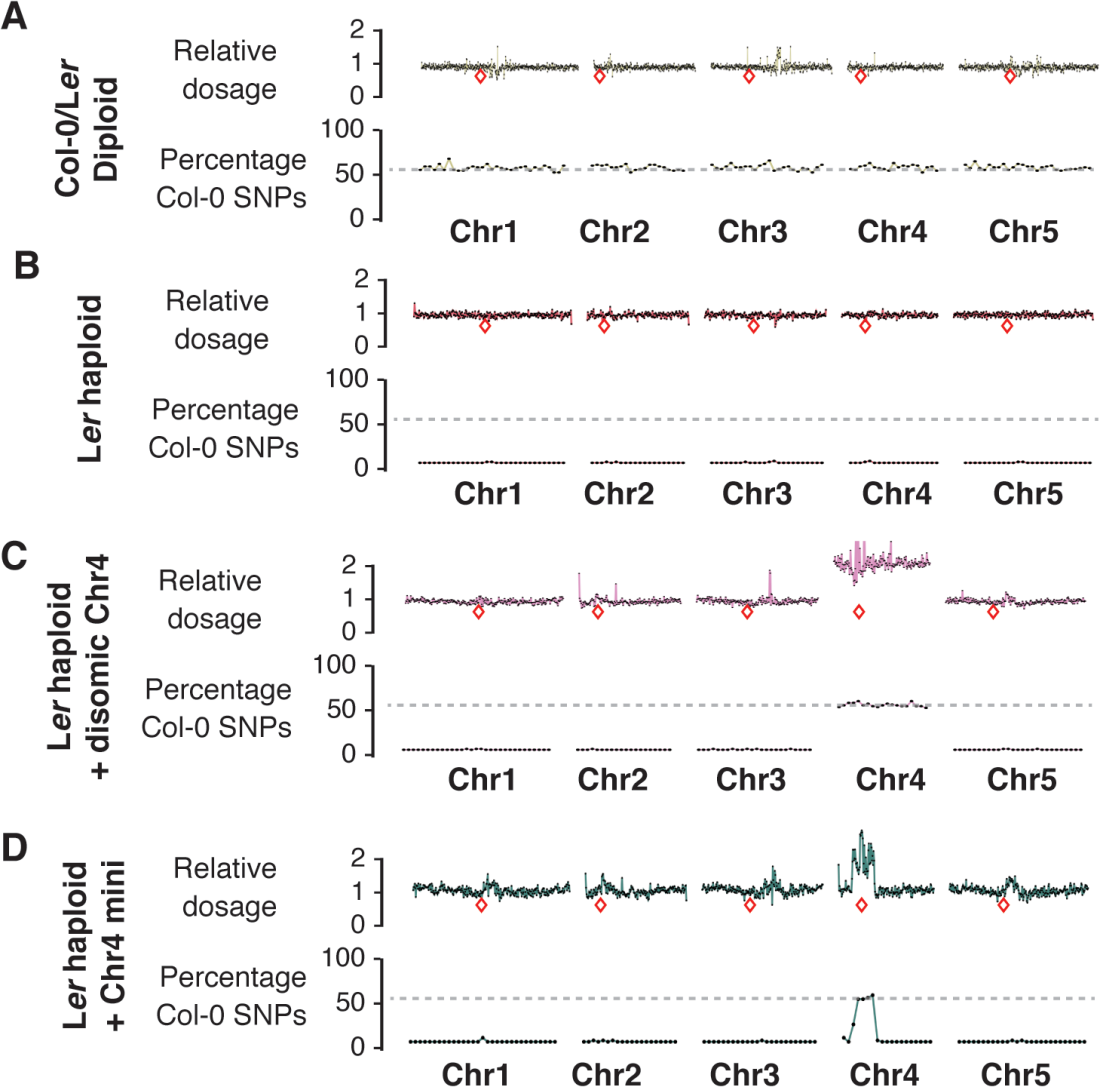
Characterization of haploid genotypes using whole-genome sequencing. (A-D): Top panels show the dosage plots for non-overlapping 100 kb bins across all five *Arabidopsis* chromosomes with the relative dosage indicated on the y-axis. The bottom panels in each section show SNP analysis based on 1 Mb bins with the percentage of Col-0 SNPs plotted. Regions with 100% Ler SNPs will have 0% Col-0 SNPs. Relative locations of centromeres are indicated by a red box. A diploid Col/L*er* hybrid control (A) is shown along with a L*er* haploid (B). Aneuploid haploids such as a haploid with disomic Chr4 (C) and a Chr4 minichromosome (D) are shown here as well.

Next, we performed whole genome sequencing on the resulting haploids to determine their genome contributions. A total of 41 glabrous plants (putative haploids based on phenotyping or flow cytometry) from haploid induction crosses were analyzed(S3 Table). On a genomic dosage plot [20, 28], true paternal haploids will appear euploid with no change in the relative copy number of each chromosome. These chromosomes, however, will carry only paternal sequences (L*er* SNPs), in contrast to a true Col-0/L*er* diploid from the cross that carries 50% Col-0 SNPs (Fig 4A). Of the 17 putative haploids from P82S crosses, 14 were euhaploids (Fig 4B). The remainders of the haploids were L*er* plants carrying, in addition, parts of the Col-0 genome: one was disomic for Chr4 (Fig 4C), one contained a Chr4 minichromosome (Fig 4D) and one was disomic Chr4 and also had a Chr5-derived minichromosome. Analyses of 18 putative haploids from G83E showed that 17 were true L*er* haploids except for one, which was a Chr4 disomic. Lastly, all 7 *glabrous* plants from the A136T cross were true L*er* haploids.

To determine whether these putative haploids would spontaneously double to produce diploids, we allowed these (nearly sterile) plants to self-pollinate. All haploids plants from the mutants P82S and G83E produced seeds albeit at very low level (20–30 seeds/plant vs. several thousand for wild-type). The seeds were normal in appearance, germinated well and produced glabrous, erecta and fully fertile offspring. Analysis of ploidy by flow cytometry revealed that the 2C peak of these plants indeed matched the position of the 2C peak of L*er gl1-1* (Fig 3G). These diploid progeny of haploid plants might have arisen via the fortuitous fusion of gametes that were carrying a complete set of five chromosomes each, as has been previously observed in mutants of Arabidopsis in which the gametes segregate without pairing [29].

Our transgenic experiments suggest that a variety of mutations in conserved residues of the CENH3 histone fold domain may result in haploid-inducers that are normal in appearance and fully fertile on self-pollination, while inducing haploids on out-crossing. Thus, haploid inducers may exist among mutagenized populations, or even among natural variants. To test this hypothesis, we analyzed a TILLING (Targeting Induced Local Lesions IN Genomes) population generated by Henikoff and Comai [30]. The mutation density of this EMS(Ethylmethane sulfonate)-treated population was about 3.89 mutations per megabase [30] per plant. In a previous screen [30] of approximately 3000 plants from this population, 4 point mutations were found in the histone fold domain. Among these, one was a silent mutation. The remaining three were A86V, R176K and W178* (W178 to stop codon). Using SIFT, A86V and W178* were predicted to be “not tolerated” and R176K to be tolerated. However, W178 is the last amino acid of CENH3 and this residue is not conserved (Fig 1). We tested the haploid inducing potential of homozygous A86V plants by pollinating them with L*er gl1-1*. The F1 seeds displayed 32% seed death, a trait found when other CENH3-based haploid inducers are crossed with wild type [11, 21, 31]. We found that 3/110 (2.7%) of the surviving F1 offspring were trichomeless, consistent with these being paternal haploids. Subsequently we created the same A86V mutation synthetically and transformed into *CENH3/cenh3-1* plants. *cenh3-1/cenh3-1* segregants expressing A86V-*CENH3* when crossed by pollen from L*er gl-1/gl1-1 CENH3/CENH3* produced 3.87% haploids. This demonstrates the equivalency of the transgenic and mutational approach. Importantly, haploid inducing lines can be derived from existing populations of plants without transgenic manipulation, simply by screening for mutations in conserved residues of the histone fold domain.

In order to determine whether our simple four-species comparison (*A*. *thaliana*, *B*. *rapa*. *S*. *lycopersicum*, and *Z*. *mays*) was somehow unrepresentative of the diversity of CENH3 in angiosperms at these 7 residues, we searched additional published plant genomes to determine whether any of these species carry the amino acid substitutions described here. We found no changes in the HFD residues in 60 published Arabidopsis ecotypes [32], but found one amino change in the hypervariable N-terminal tail (S4 Table). Our comparison of 53 angiosperm sequences from 50 different species (S1 Fig, S5 Table), revealed that 5 of our 7 investigated amino acids displayed no variation at all, while 2 (equivalent positions P82 and G173) did exhibit some diversity (S5 Fig, S6 Table). One of these substitutions (P82S) confers a haploid inducer phenotype in Arabidopsis, displaying approx. 19% seed death on outcrossing. Evidently, this same amino acid change arose and persisted in 4 different clades of angiosperms, ranging from dicots to monocots (S5 Fig, S6 Table) [33].

## Discussion

Our results on the effects of CENH3 single amino acid variation have two major implications, one basic, the other applied. On the basic side, our results reveal that some single amino acid substitutions can be as efficient as large-scale changes in producing haploid inducers. We found that altering single highly conserved amino acid residues in the histone fold domain results in fit and fertile plants that display postzygotic incompatibility and produce haploids when crossed to the wild type. Centromeres determined by point mutations in *CENH3* specify efficient chromosome inheritance in self-crosses, but lead to missegregation in an F1 hybrid when confronted with centromeres determined by the wild-type CENH3. As a result, the hybrid embryo undergoes genome elimination, producing frequent abortion (which may be due to aneuploidy-induced failure of the embryo or endosperm), and aneuploidy or haploidy among the surviving seeds.

Using human cell lines, Tachiwana et al., 2011[34] have shown that mutations in CENP-A (human CENH3) HFD loop 1 residues R80 and G81 lead to reduced CENP-A retention in the centromere. CENP-A residues L111, L128 and I132 are involved in CENH3/CENH3 interaction [34, 35]. In addition, mutation in CID (*Drosophila* CENH3) D211 also results reduced dimerization and mislocalization of the protein [36]. Although we do not have complementation data on the corresponding residues in *Arabidopsis* CENH3, three of our point mutant haploid inducers, P82S, G83E and A86V, are located immediately before the α-N-helix (Fig 1). Based on the crystal structure of CENP-A, Tachiwana et al. [34] proposed that decreased length of the CENP-A α-N-helix compared the homologous region of H3 confers loose conformation to DNA at the entrance and exit of the CENH3 nucleosomes and that the residues corresponding to the At-CENH3 P82, G83, A86 interact with DNA. The loose connection of DNA to CENP-A nucleosome may be important for centromeric function [34]. These mutations may thus alter the fundamental properties of CENH3 nucleosome thus disrupting the normal behavior of centromeric chromatin.

Further in the HFD, two of our haploid inducing mutations, A132T and A136T, reside in the CATD domain, which in human CENP-A was shown to interact with HJURP [37], a factor necessary for efficient loading of CENP-A into nucleosomes. Even though the HJURP homolog has not been identified in plants, the KNL2 protein of *A*. *thaliana* is related to the factor that recruits HJURP to the centromere [19], suggesting some conservation in CENH3 recruitment to centromeric chromatin. The deleterious post-zygotic defects observed in hybrids of these mutants to wild-type CENH3, are consistent with the possibility of defective loading.

Plants carrying the haploid-inducing point mutations described above are fully fertile, thus the gametes produced by these plants obviously carry functional centromeres. However, when encountering centromeres from wild-type plants, the mutant-derived chromosomes missegregate frequently while the wild-type derived chromosomes segregate normally [11, 20, 21]. The striking difference between embryos that inherited the mutant CENH3 from both parents, and those that inherited a mutant and a wild-type allele implies that the mutant-determined centromeres are defective in the context of the wild-type ones. A competition may be set up for some as-of-yet unidentified aspect of centromere specification, kinetochore building, or spindle attachment. Zygotic reloading of CENH3 has been suggested by observation of GFP-tagged CENH3 by Ingouff et al [38]. Defective reloading of CENH3 has been detected in developing embryos in Hordeum crosses leading to natural genome elimination [39]. Accordingly, differential loading rate or density of CENH3 or other centromeric factor between wild-type and “mutant” centromeres could explain why centromeres determined by mutant CENH3 function well in self crosses but fail in out crosses.

Our choice of highly conserved amino acids as targets for mutagenesis was largely motivated by our desire to be able to translate our results to crop species. Given the fact that our plants are viable and fully self- fertile, our results raise the question of why these particular amino acids are conserved and what, if at all, is the evolutionary significance of the outcrossing incompatibility determined by the observed changes.

Survey of natural variation found that five of the seven changes are conserved. Although no deleterious effect is apparent in our analyses, it is possible that these changes may have hidden or conditional fitness consequences. Four out of the five amino acid substitutions tested result in a penalty on outcrossing, as a large fraction (up to approximately 30%) of outcross progeny spontaneously abort, and the mutant genome is lost from among a smaller fraction of the survivors. It is possible that this outcrossing penalty alone is sufficient to explain the purifying selection of the residues at these particular positions (G83, A86, A132, A136).

Two of the residues we tested were not conserved among angiosperms. One of these substitutions (P82S) is a haploid inducer in Arabidopsis, displaying approx. 19% seed death on outcrossing. Nevertheless, this same amino acid change apparently arose and persisted in 4 different clades of angiosperms, ranging from dicots to monocots (S5 Fig, S6 Table) [33]. While we have yet to determine whether this mutation has a reproductively isolating effect in any species other than Arabidopsis, this result suggests that the mutation is well tolerated (as are P82A and P82V). In conclusion, alleles found to be HI-inducing in *Arabidopsis* have been evolutionarily successful in other plant species.

**Fig 5:**
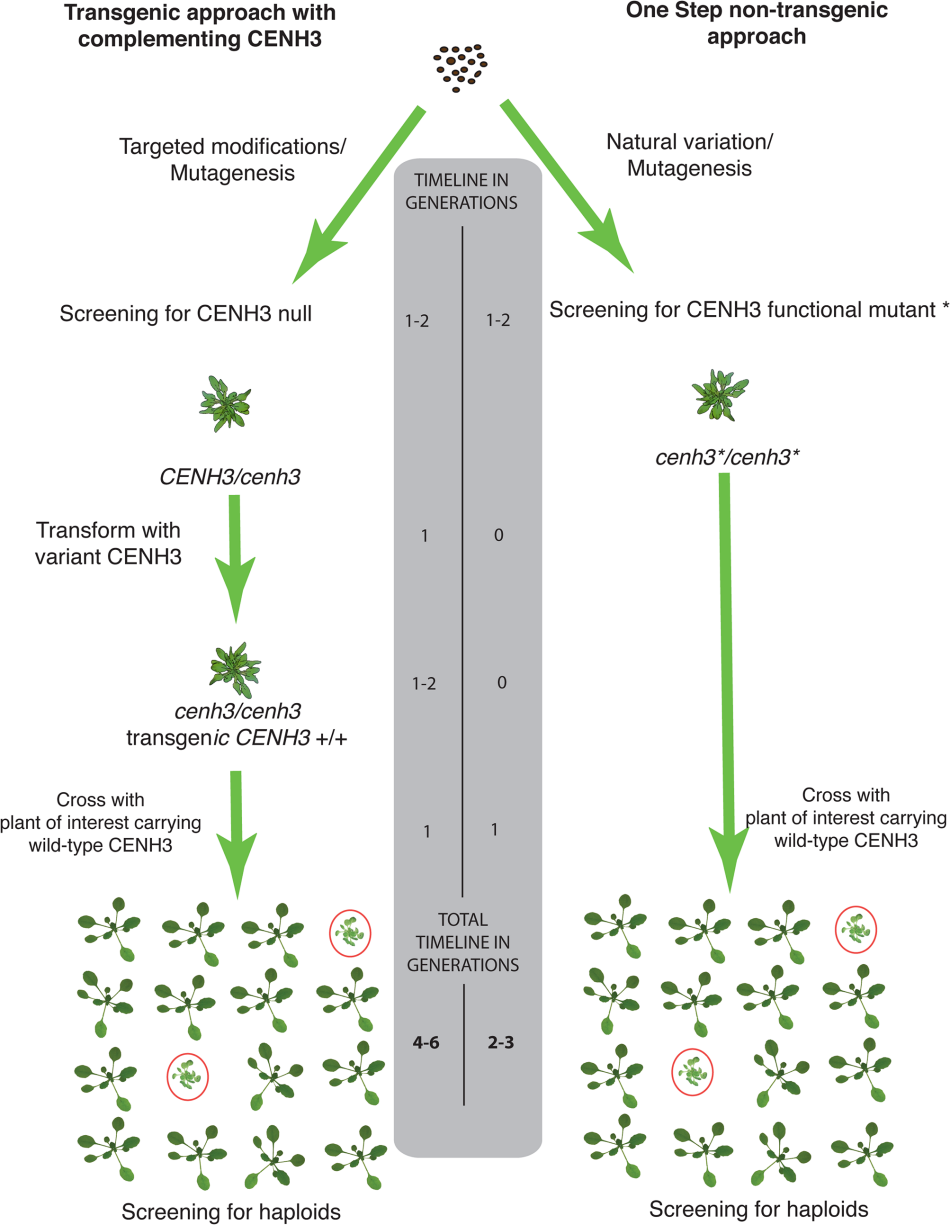
Schematic comparison of transgenic two-step vs. non-transgenic one step haploid inducers. In the first approach represented on the left, a *CENH3* knockout can be generated by CRISPR-CAS9 or identified from an EMS mutagenized population and complemented with an altered version of *CENH3*. On the right, the one step non-transgenic approach functional point mutants are identified by TILLING or from natural variation and used directly as haploid inducers. A comparison of the estimated generation times for each approach is shown in the center.

On the applied side, our findings are relevant to plant breeding. Haploids, which can be doubled to produce perfect homozygotes [21, 40–43] greatly accelerate plant breeding [40], genome assembly from sequence reads in heterozygous species[44], the production of recombinant inbred lines [45] and genetic analysis [31], but are not available for many crop species. Haploid induction through the chimeric version of CENH3 (*GFP*-tailswap) has been demonstrated in reverse breeding[46], synthetic clonal reproduction [47] and rapid QTL mapping [45] in the model plant *Arabidopsis thaliana*, underscoring the potential of this method [48]. The delayed application in crop plants, however, indicates the difficulties in engineering a system that requires combining a chimeric transgene with a knockout of the endogenous gene. A single-step, non-transgenic haploid inducer system such as described here overcomes this shortcoming (Fig 5). To extend its applicability across plant species, this study focused on amino acid residues conserved in angiosperms. It is plausible that single amino acid changes in variable or less conserved residues may have similar effects. The point mutants of *CENH3* that can produce uniparental haploids, all G:C to A:T transitions, are readily identified in existing TILLING [49] populations and so can be immediately applied to crop species, or could be induced in a single step by CRISPR-Cas9 mediated changes. Our analysis suggests that there are 47 highly conserved, EMS-mutable targets in the *CENH3* histone fold domain, of which 38 are predicted by SIFT to be “not tolerated”. Given the frequency at which we identified haploid inducers among the mutations predicted “not tolerated” by SIFT (4 out of 5 tested), our results suggest that our list of 38 mutable sites, if found to be able to complement the *cenh3-1* null (Fig 1 and S1 Table), would be excellent candidates for haploid induction.

## Materials and Methods

Cloning and transformation: Binary vector pCAMBIA-1300 (GenBank: AF234296.1) was used for cloning. The native *CENH3* promoter, 5’ UTR and 3’ UTR were cloned into this vector for earlier studies[21, 50]. This clone was used as a starting vector for our study. Cloning was done in three steps. Step 1: *CENH3* tail region with introns until first half of intron before HFD was cloned into the KpnI, XbaI site between 5’ and 3’ UTR. Step 2: fragment containing *attR*1 and *attR*2 site with *CcdB* resistance gene was cloned between the *CENH3* tail and 3’ UTR into *Bgl*I and *Xba*I site. Step 3: WT-HFD and the point mutants flanked by attL1 and attL2 were synthesized without introns through Genewiz Inc. LR recombination was done to obtain the complete *CENH3* and transformed into *E*. *coli* strain DH5α. The destination vectors were sequenced and transformed into *Agrobacterium tumefaciens* GV3101 strain and used for *Arabidopsis* transformation by conventional floral dip method [51].

### Plant growth conditions

Plants were grown in Sunshine professional growing mix # 1 (Sun Gro Horticulture) at 16h/8h light/dark cycles at 20°C in Conviron walk in chamber with a relative humidity of 50–55%.

### DNA extraction and genotyping

The plants were screened on hygromycin selection for T-DNA integration. The antibiotic resistant lines were analyzed for native *CENH3* loci by two-step genotyping method. Genomic DNA was extracted from two-week-old seedling using standard methods using CTAB buffer. First a 3 KB region specific to the native CENH3 was amplified. The first round amplification was targeted with primers flanking 2 kb upstream of the start codon of the native *CENH3* and within intron number 1 of the histone fold domain (HFD). Second round of PCR was performed with specific dCAPS primers to amplify a specific 210bp fragment flanking the site of mutation. The second round PCR product was digested with *Xba*I for overnight and resolved on 2.5% agarose gel.

### Crossing and analysis of F1 offspring

Lines carrying transgene with that were homozygous for *cenh3-1*/*cenh3-1* at the native *CENH3* loci were used as female parent in the crossing. Young flower buds were emasculated and crossed by pollen from L*er gl-1/gl1-1 CENH3/CENH3*. The seeds were harvested after three weeks. Seed death was assessed under dissection microscope. Offspring were phenotyped for glabrous (as 2 week old seedlings, under a dissecting microscope) and erecta (as 2 month old plants) traits and subsequently analyzed by flow cytometry and chromosome count

### Flow cytometry

Flow cytometric determination of genome content of the wild-type, putative haploids and double haploids were done as described [52]. Young unopened flower buds were used for nuclei isolation. The samples were chopped vigorously in chopping buffer (15mM HEPES, 1mM EDTA, 80mM KCL, 20mM NaCL, 300mM sucrose, 0.20% triton-X (for nuclei stability), 0.5mM spermine)using new razor blade (VWR Cat. #55411–050) and filtered through Falcon Blue Nylon Mesh Cell Strainer, 40 Micron (BD 352340). The flow through was then centrifuged at 500g for 7 minutes at 4°C. The nuclear pellet was then washed twice in 0.5 ml of chopping buffer. The samples were then resuspended in 0.5 ml of ice-cold-staining solution containing propidium iodide. Flowcytometric analysis was done on Becton Dickinson FACScan Flow Cytometer equipped with dual laser of 488 and 635nm wavelength and five photodetectors. The data was acquired and analyzed using the Cellquest software.

### Chromosome count

Chromosome count of the wild-type, haploids and double haploids were performed as described in[53]. Young flower buds were fixed in Carnoy’s fixative overnight and washed three times in 70% ethanol. Mature flower buds were dissected out and young flower buds at the apex of the inflorescence were selected for further treatment. The young flower buds were then digested with 3% cellulase and 3% pectinase for three hours. Following digestion, the flower buds were dissected under a microscope to isolate anthers. After removing the debris, the anthers were teased to release the cells followed by addition of 60% acetic acid. The cells were spread using 60 ul of ice-cold 3:1 Ethanol acetic acid. The slides were allowed to dry and DAPI (1 um) stain was added. The slides were visualized under Zeiss LSM 710 confocal microscope and the image were acquired using Zeiss image processing software.

### Imaging of haploids and control

Seedlings were allowed to germinate on MS plates for two weeks. The presence/absence of trichomes on the first true leaves were observed and imaged using a dissection microscope equipped with Carl Zeiss Axiocam color HRc camera for imaging. The image was acquired with Zeiss software. To observe the fertility phenotype, plants were allowed to grow for 6–7 weeks. Wild-type control and haploid plant of same age grown under same conditions were imaged using Kodak easyshare 14MP camera equipped with AF3x optical Aspheric lens 32mm-96 mm.

### Whole genome sequencing

DNA extraction was performed using Nucleon PhytoPure DNA extraction kit (GE Healthcare Life Sciences Inc.). DNA was sheared to 300–400 bp fragments using Covaris E220 sonicator under following settings: Peak incident power 175, duty factor 5%, cycle per burst 200, treatment time 60s at 7°C. Library prep for illumina sequencing was done using standard NEB next DNA Library prep and BIOO Scientific NEXTFlex-96 adapters were used. Samples were pooled and sequenced on MiSeq 2500 for 50bp single reads. The resulting reads were further analyzed as described in[28].

### Pollen fertility assay

Pollen fertility assay was performed using standard staining protocol as described in[54]. Anthers from the unopened flower bud were dissected and stained with the staining solution containing 1% each of Malchite green, Acid fuchsin and Orange G. Images were acquired using Nikon eclipse E600 microscope equipped with Nikon Digital sight DS5C camera under 10X magnification using NIS Elements software version F3.0.

### Alignment of protein sequences

Protein sequences were downloaded from GeneBank (http://www.ncbi.nlm.nih.gov/genbank/). The CENH3/CENP-A annotated tail regions were removed from the sequences and the histone fold domains were aligned using Geneious software version 6.0.5[55] using the ClustalW alignment method with the following conditions: Cost matrix: Blosum, Gap open cost: 10 and Gap extend cost: 0.1.

## Supporting Information

S1 Fig Multiple amino acid sequence alignment of CENH3 from 50 plant species using global alignment with blosum scoring matrix. *Nicotiana tabacum*, *O*.*alta* and *O*.*minuta* are allotetraploid species with two genomes that carry two different *CENH3*. The N-terminal tail and histone fold domain are marked at the top of the alignment. Inset blue box shows the amino acid similarity index used.
(TIF)

S2 Fig Ethidium bromide stained gel showing the strategy for genotyping the *CENH3* locus in plants carrying transgenic *CENH3* with point mutations. (A) The first round amplification is targeted with primers flanking 2 kb upstream of the start codon of the native *CENH3* and within intron number 1 of the histone fold domain (HFD). The synthetic construct used for transgenic point mutants do not contain any introns in the HFD. (B) The second round of PCR was performed on the PCR product from (A) using standard genotyping procedure for the *cenh3-1* allele to determine the genotype for the native *CENH3* locus of transgenic plants.
(TIF)

S3 Fig Rosette leaf phenotypes of transgenic plants from this study. (A) Wild-type phenotype of a Col-0 plant (B-H) Transgenic *cenh3-1/cenh3-1* complemented with *CENH3* point mutations. P82S, G83E, A86V, A132T and A136T (B-D, F, G) are haploid inducers while P102S and G173E (E, H) are non inducers.
(TIF)

S4 Fig Pollen viability assay from dissected anthers. (A) Anther from wild-type Col-0. (B-I) Anthers from transgenic *cenh3-1/cenh3-1* mutant complemented with various *CENH3* variants. Pollen grains that are stained red are viable while inviable pollen grains are stained green. Semi-sterile pollen from the anther of *cenh3-1/cenh3-1 GFP*-tailswap (B) only contain a few viable pollen while the anthers from transgenic point mutants (C-I) appear viable.
(TIF)

S5 Fig Amino acid sequence alignment of the CENH3 histone fold domain from six rice species. The first residue of the histone fold domain is highlighted within a magenta box. *O*.*alta* sequences from its C and D genomes are within a blue box while *O*.*minuta* sequences from its B and C genomes are within an orange box. The alignment was based on the blosum scoring matrix and an inset red box shows the similarity index in this alignment.
(TIF)

S1 Table Conserved amino acids across *Arabidopsis thaliana*, *Brassica rapa*, *Solanum lycopersicum*, and *Zea mays*. CENH3 histone fold domain that can be mutated to same amino acid by G to A or C to T transition. Columns 2–4 show the triplet codons while columns 6 show the corresponding amino acids. The EMS-inducible G to A or C to T transitions and corresponding change to amino acid codon is shown in columns 8 and 9.
(PDF)

S2 Table SIFT prediction of protein function for substitutions of amino acids in Arabidopsis CENH3. Threshold for intolerance was set at 0.05. Amino acid color code: nonpolar (black), uncharged polar (green), basic (red) and acidic (blue). Uppercase letters denote amino acids that appear in the alignment, lower case letters indicate amino acids that did not appear in other sequences in the alignment. 'Seq Rep' refers to the fraction of aligned sequences that contain the same or similar amino acids. A low ratio indicates the position is either severely gapped or unalignable and has little information. Predictions made at these positions are not very accurate. The 47 EMS-inducible changes in conserved amino acids identified in this study are highlighted in yellow.
(PDF)

S3 Table Characteristics of all haploids analyzed by whole genome sequencing from this study. Each row represents the haploid individual (with unique HAP ID's), the parental haploid inducer line as well as the observed chromosome content based on dosage and SNP analysis. The last column describes the proposed karyotype of each individual.
(XLSX)

S4 Table Table showing the single nucleotide polymorphisms (SNPs) across the *CENH3* gene from 60 Arabidopsis accessions. Only polymorphisms within the coding region are represented here. * indicates an amino acid change as a result of the SNP. SNPs #1 and #2 were found in following accessions; TueV13, TueWa1-2, TueScha9, ICE173, ICE191, ICE102, Mer-6, Ped-0, ICE50, ICE49, Vash-1, Lag2.2 ICE63, Kastel-1, ICE138. SNP #3 was found in following accessions; ICE72, ICE61, ICE60, Yeg-1, ICE29. SNP #4 was identified in following accessions: Nie1-2, ICE216, ICE212, ICE213, ICE119, ICE112, Bak-2.
(PDF)

S5 Table Table showing the conservation of amino acid residues within the CENH3 histone fold domain across 53 angiosperm CENH3 sequences. Column 4 indicates the amino acid changes tested in this study and column 5 indicate if that particular mutation can act as a haploid inducer (highlighted in green) or not (highlighted in magenta).
(PDF)

S6 Table Analysis of amino acid conservation from 50 angiosperm species. (A) Variant amino acid residues from different species at position corresponding to residue 82 from *A*. *thaliana*. (B) Variant amino acid residue from different species at position corresponding to residue 173 from of *A*. *thaliana*. Highlighted in yellow (A) are the species that carry P82S polymorphisms. *O*. *alta* and *O*. *minuta* are alloteraploid rice species that carry CCDD and BBCC genomes respectively.
(PDF)
