# Supplementary figures and images for: Point Mutations in Centromeric Histone Induce Post-zygotic Incompatibility and Uniparental Inheritance
Source: PLoS Genet. 2015 Sep 9;11(9):e1005494. doi: 10.1371/journal.pgen.1005494 (PMC4564284; doi:10.1371/journal.pgen.1005494)

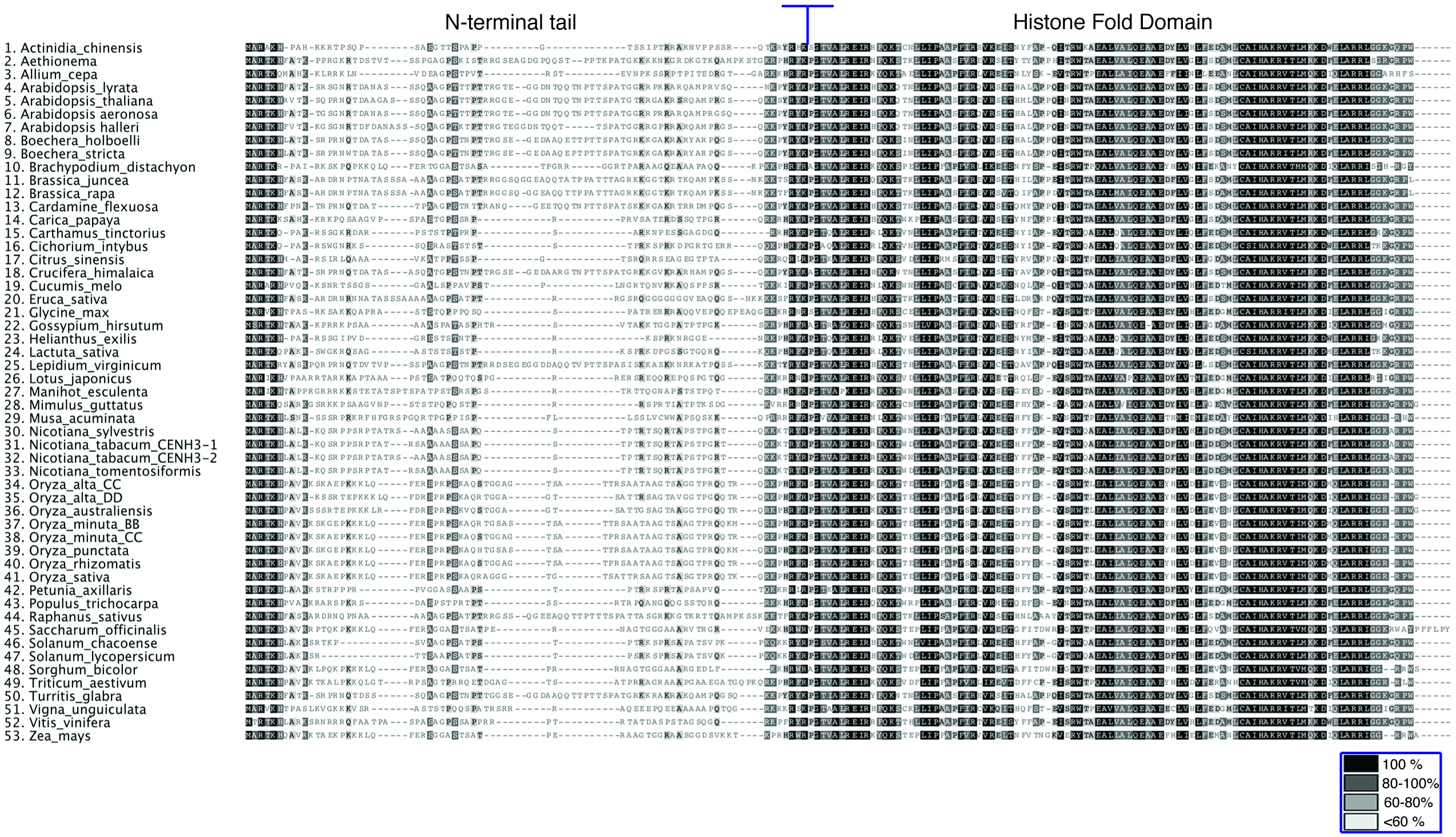

Supplement: S1 Fig — Nicotiana tabacum, O.alta and O.minuta are allotetraploid species with two genomes that carry two different CENH3. The N-terminal tail and histone fold domain are marked at the top of the alignment. Inset blue box shows the amino acid similarity index used. (TIF) [file pgen.1005494.s001.tif]

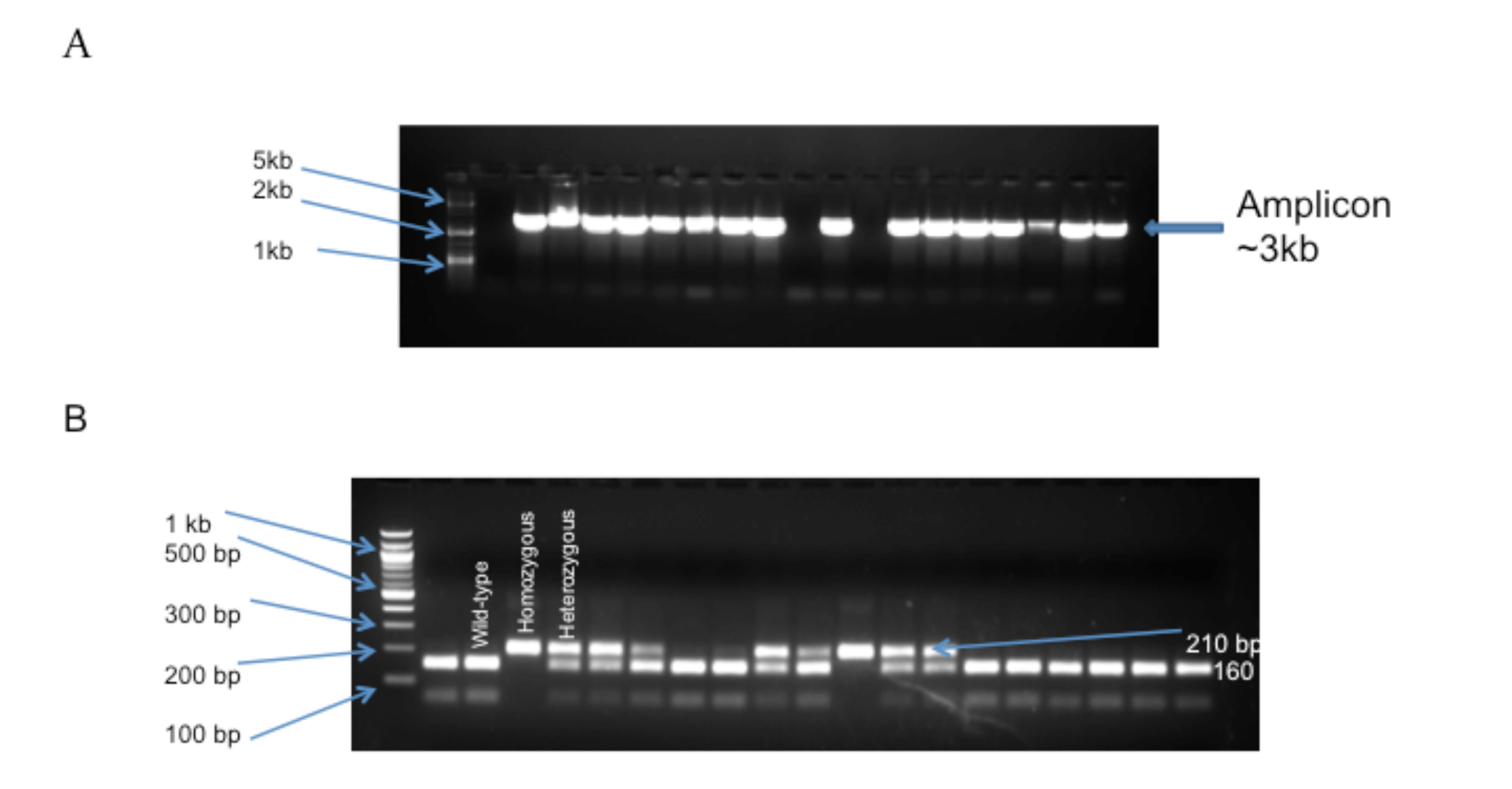

Supplement: S2 Fig — (A) The first round amplification is targeted with primers flanking 2 kb upstream of the start codon of the native CENH3 and within intron number 1 of the histone fold domain (HFD). The synthetic construct used for transgenic point mutants do not contain any introns in the HFD. (B) The second round of PCR was performed on the PCR product from (A) using standard genotyping procedure for the cenh3-1 allele to determine the genotype for the native CENH3 locus of transgenic plants. (TIF) [file pgen.1005494.s002.tif]

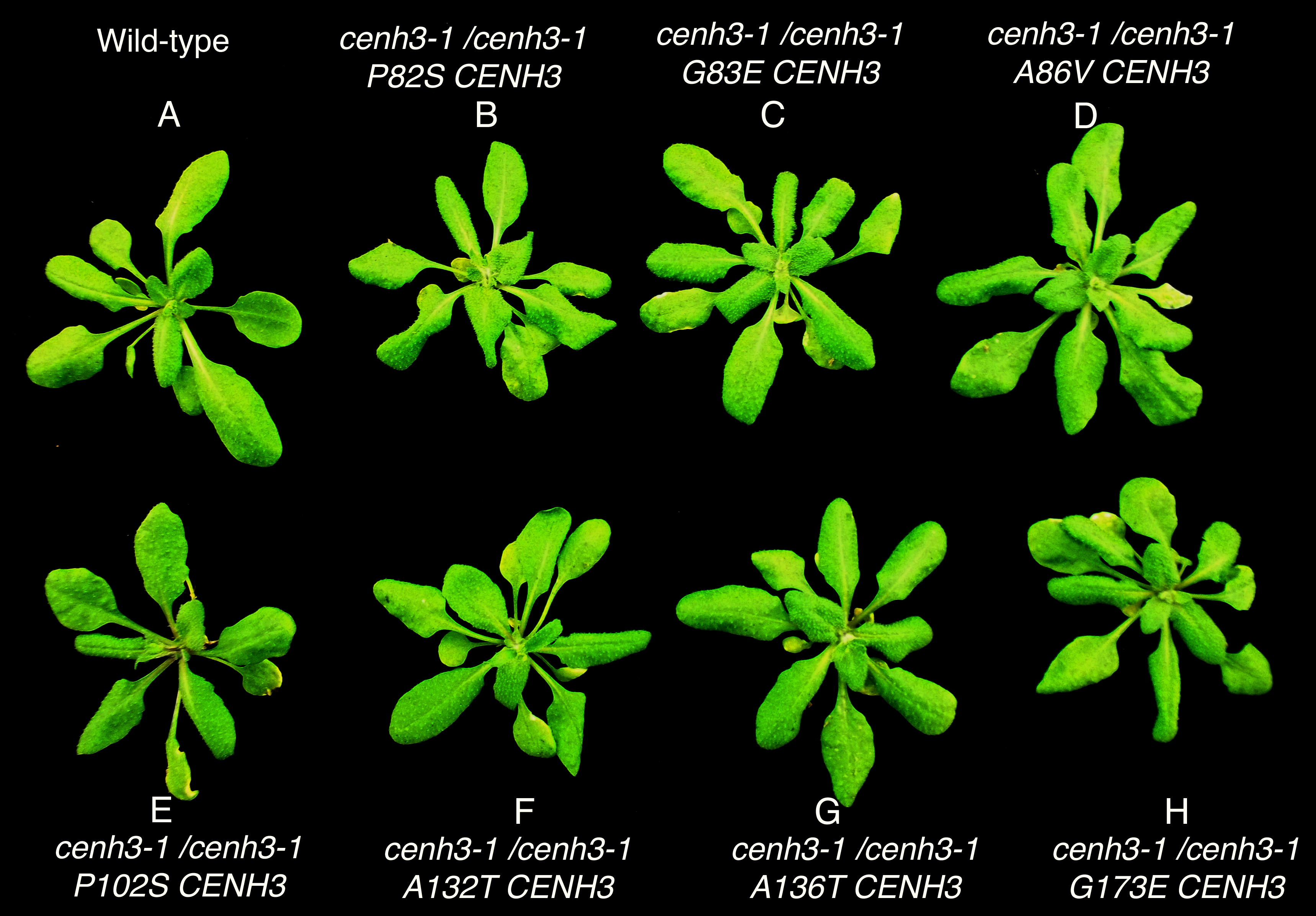

Supplement: S3 Fig — (A) Wild-type phenotype of a Col-0 plant (B-H) Transgenic cenh3-1/cenh3-1 complemented with CENH3 point mutations. P82S, G83E, A86V, A132T and A136T (B-D, F, G) are haploid inducers while P102S and G173E (E, H) are non inducers. (TIF) [file pgen.1005494.s003.tif]

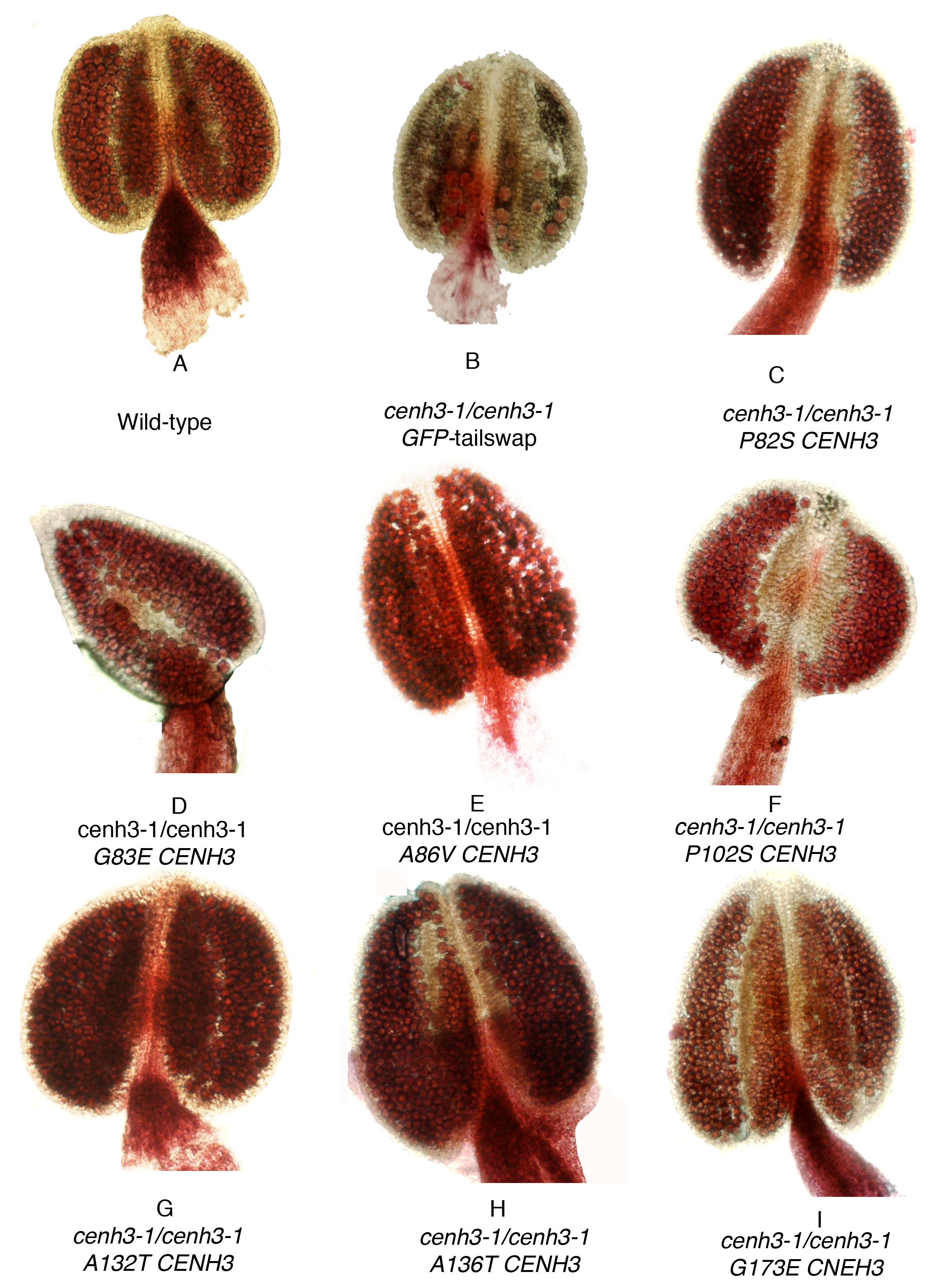

Supplement: S4 Fig — (A) Anther from wild-type Col-0. (B-I) Anthers from transgenic cenh3-1/cenh3-1 mutant complemented with various CENH3 variants. Pollen grains that are stained red are viable while inviable pollen grains are stained green. Semi-sterile pollen from the anther of cenh3-1/cenh3-1 GFP-tailswap (B) only contain a few viable pollen while the anthers from transgenic point mutants (C-I) appear viable. (TIF) [file pgen.1005494.s004.tif]

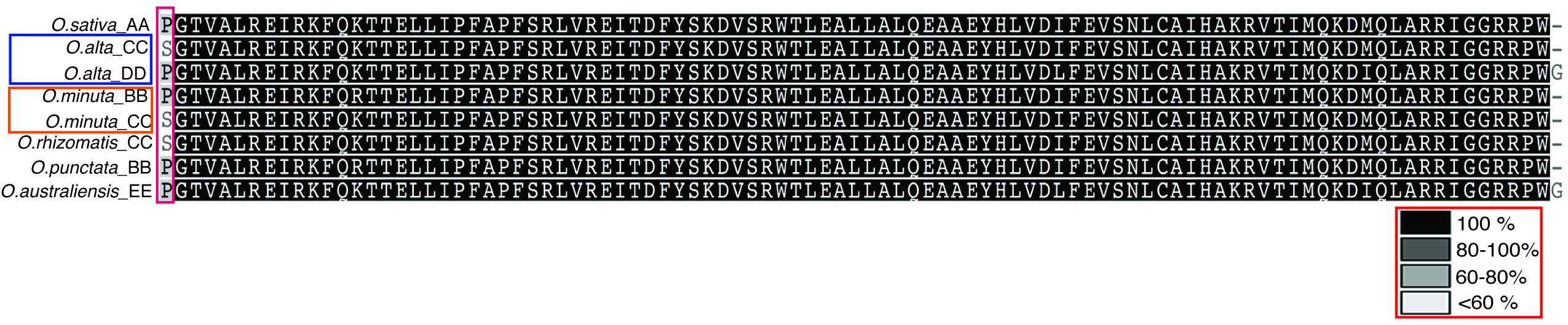

Supplement: S5 Fig — The first residue of the histone fold domain is highlighted within a magenta box. O.alta sequences from its C and D genomes are within a blue box while O.minuta sequences from its B and C genomes are within an orange box. The alignment was based on the blosum scoring matrix and an inset red box shows the similarity index in this alignment. (TIF) [file pgen.1005494.s005.tif]
