# Supplementary material for: Point Mutations in Centromeric Histone Induce Post-zygotic Incompatibility and Uniparental Inheritance
Source: PLoS Genet. 2015 Sep 9;11(9):e1005494. doi: 10.1371/journal.pgen.1005494 (PMC4564284; doi:10.1371/journal.pgen.1005494)
Supplement: S1 Table — CENH3 histone fold domain that can be mutated to same amino acid by G to A or C to T transition. Columns 2–4 show the triplet codons while columns 6 show the corresponding amino acids. The EMS-inducible G to A or C to T transitions and corresponding change to amino acid codon is shown in columns 8 and 9. (PDF) [file pgen.1005494.s006.pdf]

Mutation number	*A.thaliana*	*B. rapa*	*S. lycopersicum*	*Z. mays*	Amino acid position in Arabidopsis	Original amino acid	Mutated amino acid	Mutated codon
1	CCA	CCT	CCA	CCA	82	P	S	TCA
2	CCA	CCT	CCA	CCA	82	P	L	CTA
3	GGA	GGA	GGG	GGG	83	G	R	AGA
4	GGA	GGA	GGG	GGG	83	G	E	GAA
5	ACC	ACC	ACA	ACT	84	T	I	ATC
6	GCT	GCC	GCA	GCG	86	A	T	ACT
7	GCT	GCC	GCA	GCG	86	A	V	GTT
8	GAG	GAG	GAA	GAG	89	E	K	AAG
9	CTT	CTT	CTT	CTC	100	L	F	TTT
10	CCG	CCT	CCA	CCC	102	P	S	TCG
11	CCG	CCT	CCA	CCC	102	P	L	CTG
12	GCT	GCC	GCT	GCG	104	A	T	ACT
13	GCC	GCT	GCT	GCG	104	A	V	GTC
14	CGT	CCT	CCT	CGC	124	R	C	TGT
15	CGT	CGT	CGT	CGC	124	R	H	CAT
16	GCT	CGT	CGT	GCA	127	A	T	ACT
17	GCT	GCT	GCT	GCA	127	A	V	GTT
18	GAA	GAA	GAG	GAA	128	E	K	AAA
19	GCT	GCT	GCG	GCC	129	A	T	ACT
20	GCT	GCT	GCG	GCC	129	A	V	GTT
21	GCT	GCT	GCT	GCG	132	A	T	ACT
22	GCT	GCT	GCT	GCG	132	A	V	GTT
23	GAG	GAG	GAG	GAG	135	E	K	AAG
24	GCG	GCG	GCT	GCA	136	A	T	ACG
25	GCG	GCG	GCT	GCA	136	A	V	GTG
26	GCA	GCT	GCT	GCA	137	A	T	ACA
27	GCA	GCT	GCT	GCA	137	A	V	GTA
28	GAA	GAA	GAA	GAA	138	E	K	AAA
29	TCA	GCG	GCA	GCG	148	S	T	ACA
30	TGT	TGC	TGT	TGT	151	C	Y	TAT
31	GCT	GCT	GCT	GCC	152	A	T	ACT
32	GCT	GCT	GCT	GCC	152	A	V	GTT
33	CAT	CAC	CAT	CAT	154	H	Y	TAT
34	GCA	GCA	GCG	GCC	155	A	T	ACA

35	GCA	GCA	GCG	GCC	155	A	V	GTA
36	CGT	CGT	CGT	CGT	157	R	C	TGT
37	CGT	CGT	CGT	CGT	157	R	H	CAT
38	GTT	GTT	GTT	GTC	158	V	I	ATT
39	ACT	ACT	ACA	ACA	159	T	I	ATT
40	ATG	ATG	ATG	ATG	161	M	I	ATA
41	GAC	GAT	GAT	GAC	164	D	N	AAC
42	GCA	GCA	GCT	GCA	168	A	T	ACA
43	GCA	GCA	GCT	GCA	168	A	V	GTA
44	GGA	GGA	GGA	GGA	172	G	R	AGA
45	GGA	GGA	GGA	GGA	172	G	E	GAA
46	GGA	GGA	GGA	GGA	173	G	R	AGA
47	GGA	GGA	GGA	GGA	173	G	E	GAA
