# Supplementary material for: Point Mutations in Centromeric Histone Induce Post-zygotic Incompatibility and Uniparental Inheritance
Source: PLoS Genet. 2015 Sep 9;11(9):e1005494. doi: 10.1371/journal.pgen.1005494 (PMC4564284; doi:10.1371/journal.pgen.1005494)
Supplement: S2 Table — Threshold for intolerance was set at 0.05. Amino acid color code: nonpolar (black), uncharged polar (green), basic (red) and acidic (blue). Uppercase letters denote amino acids that appear in the alignment, lower case letters indicate amino acids that did not appear in other sequences in the alignment. 'Seq Rep' refers to the fraction of aligned sequences that contain the same or similar amino acids. A low ratio indicates the position is either severely gapped or unalignable and has little information. Predictions made at these positions are not very accurate. The 47 EMS-inducible changes in conserved amino acids identified in this study are highlighted in yellow. (PDF) [file pgen.1005494.s007.pdf]

Predict Not Tolerated	Position	Seq Rep	Predict Tolerated
y w v t s r q p n l k i h g f e d c a	**1M**	0.58	M
w h y d f n r q m e k c p g l s i	**2A**	0.58	T V A
c w f m d i y v h s p g n l t a e k Q	**3R**	0.58	R
g w y h d r n f q e k c m p s l A I	**4T**	0.58	V T
c w d f m y i g p s h n l a t e q V R	**5K**	0.58	K
c w f m d i y v p g s l a t n e k	**6H**	0.59	Q R H
	**7R**	0.58	c w d P M e k Q N g R i T s V A h L F Y
w h y d f n r q e m k c g s l i	**8V**	0.59	T P V A
w	**9T**	0.59	c f m y H I P L V g n R Q d T A S K e
w f d y i v h g l n s t q P C	**10R**	0.60	E M A K R
w y f c	**11S**	0.61	m i H v l P G N q R d T A E S K
w y f c	**12Q**	0.64	m h I p v L G d N A T Q E K S R
w y f c	**13P**	0.65	m h i v l q G N T A R e D S P K
w y f c m	**14R**	0.68	H i v P L n G Q D T s E A K R
w y f	**15N**	0.67	c m H i v l P G Q R D N T s A E K
w y	**16Q**	0.66	f c m h i V L G t N D a Q P R S e K
w y f c	**17T**	0.60	h M I l P V G N Q d R S A T e K
w y c	**18D**	0.60	F m h i l V P G R T N Q S A E D K
w y f c m h	**19A**	0.62	v l I n P R D G T S E Q K A
	**20A**	0.55	c w p m D e q k n r G I S H V T f A L Y
w y f c	**21G**	0.61	m h i l V P d T R N S K Q E A G
w	**22A**	0.60	c y m h F i V l P G n R q T d S A K E
w y f c m i	**23S**	0.63	v l g H d P N T Q R K A E S
w y f c h	**24S**	0.66	i M v l q P G D N e T A K R S
w y f c m h	**25S**	0.67	I l n r G q d P V K T E A S
w y f	**26Q**	0.64	C m h i V L r N G P D T S k Q E A
w y f c m h	**27A**	0.62	i l V d Q N P R k T S E G A
w y c m h v l	**28A**	0.64	F n I G P R D Q e T K S A
w y f c	**29G**	0.62	m h I p l V n q D T k E R A S G
w y f c m h i v l	**30P**	0.64	N R D e G A Q S K T P
w y f c m h	**31T**	0.66	i l V n G D R e K P Q A T S
w y c m h v l I	**32T**	0.66	F q G P R D e N K S A T
w y f c	**33T**	0.66	m i H v l g d Q P R N e K A S T
w y f c m h i l	**34P**	0.70	n d V Q G E A T R K S P
w y f c	**35T**	0.73	M H i v l P G n D Q R k A E S T
w y f c	**36R**	0.74	m h i v L P G N D Q S A e T K R
w y f c	**37R**	0.77	m h i l V n P T d G Q A S E K R
w y f	**38G**	0.78	c M H I V L P N R d T G Q S K E A
w y c	**39G**	0.78	F m h I p v L N D Q R T G K A E S
w y f	**40E**	0.77	c m h i V L P G n R Q d T S A K E
w y f c m h i	**41G**	0.76	v l P r N Q D A E K S T G
w c f	**42G**	0.76	Y M i H L V P d N Q R T K S A E G
w y f	**43D**	0.78	c m H I P V l G N R T Q D S A K E
w y f	**44N**	0.78	C m h i v P l G T R Q D N S K E A
w y f c	**45T**	0.79	h M v I L P n G d R Q K E S A T
w y f c	**46Q**	0.78	m h i l V P G n d T R A Q S e K
w y f	**47Q**	0.78	C m h I v l P G n R T d Q S A K E
w y f m h i l	**48T**	0.80	C G q d N P R V E A S K T
w y f	**49N**	0.80	C m h I v L G P R Q N d S T A K E
w y f	**50P**	0.82	C m h I l V G d T N Q A R S K E P

Predict Not Tolerated	Position	Seq Rep	Predict Tolerated
wy f c	**51T**	0.82	m h I P L V n G q d R A E S K T
wy f c	**52T**	0.80	m h i v L P G n d Q R S A K T E
wy	**53S**	0.80	f c M H i v P L G n q R D T A S K E
wy f c m h i	**54P**	0.81	v l n Q D S e R T K A G P
w h f y mi c n q d e k	**55A**	0.81	L P T R V G S A
wc	**56T**	0.84	y f m H i P V L G N Q d R s A e T K
c f my h i l v	**57G**	0.86	d W k e N Q R P T S A G
w y f	**58T**	0.88	c m h i V L P N G Q R T D A S K E
wy f c m	**59R**	0.87	i H l V P N Q D T A E S G R K
wy f c mh	**60R**	0.87	i v l G N D P T E S Q A R K
wc	**61G**	0.80	m h y F I v L q d P N T K e R S A G
W y f	**62A**	0.86	c m h i L V P G N R Q T D S A K E
w y c	**63K**	0.87	F M H I l V P n G T D R Q A S E K
wy	**64R**	0.88	f c m h I v l P G N T d Q R S A K e
wy f c mh	**65S**	0.88	i L V N d R Q G P E S K T A
wy f c	**66R**	0.88	M H i l V P G N T D Q R S A E K
wy	**67Q**	0.88	c F m H i V L P G N R T D Q S A K E
wy f c m	**68A**	0.88	H I l V N d R G Q K E P T S A
w y f	**69M**	0.86	c M h i P V L G n R T Q D S A K E
wf c mi Y v l H	**70P**	0.85	G N R E D K T Q A S P
wy f c	**71R**	0.90	h M l I V P N G A D T S R K E Q
wy f c mh	**72G**	0.88	I l V P D R N K E A Q S T G
wy f c	**73S**	0.87	h M I v L P n d G R Q T A S E K
w y f	**74Q**	0.87	c m H I l P V G N R t D a S Q K E
c wf md y i v s l t A Q P E N G	**75K**	0.95	H R K
c wf d mi y p h n l t a e q G S V	**76K**	0.98	R K
wf c y h i l n d	**77S**	0.98	M q V e G T S A R K P
c wd mg n i e	**78Y**	0.98	s v q P a l T K F Y R H
y wv t s q p n ml k i h g f e d c a	**79R**	1.00	R
c mp q e k r i d t g v a h l S	**80Y**	1.00	W N F Y
c wd f mi y v g p s h n a l t e q	**81R**	1.00	K R
w h y f mi n q r d e k c v t g L S	**82P**	1.00	A P
y wv t s r q p n ml k i h f e d c a	**83G**	1.00	G
w h y f r d q me c k n g p l i s A V	**84T**	1.00	T
g w h y d n q f s e c p mt I K R A	**85V**	1.00	L V
y w v t s r q p n ml k i h g f e d c	**86A**	1.00	A
y wv t s r q p n mk i h g f e d c a	**87L**	1.00	L
c wd f mi y v g p s h n a l t e Q	**88K**	1.00	K R
y wv t s r q p n ml k i h g f d c a	**89E**	1.00	E
y wv t s r q p n ml k h g f e d c a	**90I**	1.00	I
c wf d mi y v s h g p n l a t e q K	**91R**	1.00	R
c wd mi v g p s Y t l	**92H**	1.00	F e N A Q H R K
h n k r q d g e p c t s a mv i w	**93F**	1.00	L F Y
y wv t s r p n ml k i h g f e d c a	**94Q**	1.00	Q
c wf d mi y v p h g n l t e q S	**95K**	1.00	A R K
wf my c h i l r v p k d A G E	**96Q**	1.00	N Q T S
	**97T**	1.00	d m e k q P n C g r i W S h l A F V Y T
wc f y mi v l p g t a H	**98N**	1.00	R S Q K N E D
d h g n e c s r k wq t y a v mi F	**99L**	1.00	P L
d h g n e c s wr k y p q t a f mi V	**100L**	1.00	L

Predict Not Tolerated	Position	Seq Rep	Predict Tolerated
h d w n e c p g r q s k y t a f M V	**101I**	1.00	L I
w f m c y d i h n v e t	**102P**	1.00	g L S Q k A R P
	**103A**	1.00	C w d P m e q n g R I t S K v h A I F Y
d h n w y e r c g k q p S	**104A**	1.00	t f M v I A L
w h y f m i r q e d l k n v g C T A	**105S**	1.00	S P
y w v t s r q p n m l k i h g e d c a	**106F**	1.00	F
g w h y d n r f	**107I**	1.00	k e p C M Q S t L A I V
y w v t s q p n m l k i h g f e d c a	**108R**	1.00	R
w	**109E**	1.00	d p m k n g r s i T h A F C Q E Y V L
h w d q n r g e p k c s y f m t a l	**110V**	1.00	I V
c w d f m i y v g s p h n a l t e q	**111R**	1.00	K R
w f y i h l v r p n g t M k C	**112S**	1.00	A D Q S E
h w q d p n e r c g k s y m a f T L	**113I**	1.00	V I
w h f y m r i e l q d k v p n g	**114T**	1.00	C S A T
	**115H**	1.00	c w p M D E k g Q R N i t S v A H L F Y
	**116M**	1.00	c w M p D G N Q I r k E T S V H A L F Y
c w d p e k n q g s	**117L**	0.99	h a R T M I V Y F L
w y f m h	**118A**	0.99	i C p V N R G d Q T L E K A S
w c y h i v M q	**119P**	0.84	R L N e D F G K A T S P
y f c	**120P**	0.84	W m H i v l G q T N R S P D E A K
w f c Y i M	**121Q**	0.98	H v P G N R A S T L K Q D E
c w m k n r t s P h E	**122I**	0.92	A G F D L Q Y I V
w c y	**123N**	0.88	F m h i p v r Q L D a K G N E T S
y w v t s q p n m l k i h g f e d c a	**124R**	1.00	R
k q h n r d g e p c t s a m v i l	**125W**	1.00	Y F W
w f c m y i h l v g p d n a	**126T**	1.00	r E K S Q T
w h y f m i q r n d e c v K	**127A**	1.00	T L G P S A
w c f y m i v l p r g k T	**128E**	1.00	A N D H S Q E
w h y f i m n q r d e l k c v t p G S	**129A**	1.00	A
h d w n e p q c r g s k y t a f m	**130L**	1.00	V I L
h d n e w c p s r k y a G f	**131V**	1.00	T Q I M V L
w h y f i m n q r d e k l v t p g S C	**132A**	1.00	A
d h g n e c w s y r k q t a f M P	**133L**	1.00	V I L
m w f c i y v t l s a p n e d r k g H	**134Q**	1.00	Q
w c m f y i h l v r n g p s k a q d T	**135E**	1.00	E
y w v t s r q p n m l k i h g f e d c	**136A**	1.00	A
w h y f m i r q c e l d k n v p g	**137A**	1.00	T S A
y w v t s r q p n m l k i h g f d c a	**138E**	1.00	E
	**139D**	1.00	C w p M E k q D n g r I T s v h A I F Y
w p d e k q n g r t s i a v l M	**140Y**	1.00	C H F Y
d g h n e c s w y r k p q t a f V M I	**141L**	1.00	L
h w d g n q r y e k s p f m t a l C	**142V**	1.00	I V
w	**143G**	1.00	f c y m i v l p R T Q a H K S E N G D
d h g n e c s w r k y p q t a f V	**144L**	1.00	M I L
h n d k r g e q c p s t a m v w i y	**145F**	1.00	L F
c w m f i y l v r h t p n a k Q G D	**146S**	1.00	S E
w y f c h i p l M t q G N A	**147D**	1.00	S K R E V D
w h y f m i r q d e l n k v p	**148S**	1.00	C G T S A
	**149M**	1.00	c W p d e q k g r t i s a V H M f l Y N
d g n c e s w r k p q t a f v i H Y M	**150L**	1.00	L

Predict Not Tolerated	Position	Seq Rep	Predict Tolerated
k h q e n w r m d s t p y i v f a g L	**151C**	1.00	C
w h y f m i q r d n e c l k v p g T S	**152A**	1.00	A
h d w n e c p g q r s k y t a f m V	**153I**	1.00	L I
y w v t s r q p n m l k i g f e d c a	**154H**	1.00	H
w h y f m i n q r d e l k c v t p s G	**155A**	1.00	A
c w f d m i y v g p s h l a t e q	**156R**	1.00	N R K
y w v t s q p n m l k i h g f e d c a	**157R**	1.00	R
h w d n g q r e y k s p c f m t a l I	**158V**	1.00	V
y w v s r q p n m l k i h g f e d c a	**159T**	1.00	T
h d w n e p c q r g s k y t a f M	**160L**	1.00	V L I
d h n e c s k g r w p q t y a v f i L	**161M**	1.00	M
c w d f m i y v g s	**162R**	1.00	h n a l e T P R K Q
c w f d m i y v g s p h n a l t e q	**163K**	1.00	R K
y w v t s r q p n m l k i h g f e c a	**164D**	1.00	D
d h g n e c s r k p q t y a v W	**165F**	1.00	L M F I
c f m i y l v r g t n s p a k W D	**166E**	1.00	H E Q
d h n g e c s w r k y p q t a f m i V	**167L**	1.00	L
w h y f m i q r n d e c k l v p s g T	**168A**	1.00	A
y w v t s q p n m l k i h g f e d c a	**169R**	0.98	R
y w v t s q p n m l k i h g f e d c a	**170R**	0.98	R
d h g n e c s w r k y p q t a f m v	**171L**	0.98	L I
w f	**172G**	0.98	m y i h c l q e v d p k n T R S a G
y w v t s r q p n m l k i h f e d c a	**173G**	0.97	G
c w f d y	**174K**	0.71	M v g p s h n a l t q I E R K
w h y f i m q c l e n d k v t p s R	**175G**	0.42	A G
c w f d m i y v p s h l n a t e G	**176R**	0.51	K Q R
c w f d m y i v h g n s t a e Q k L	**177P**	0.54	R P
h q k n r d e g p c t s a m v i l y F	**178W**	0.47	W
