# Supplementary material for: Point Mutations in Centromeric Histone Induce Post-zygotic Incompatibility and Uniparental Inheritance
Source: PLoS Genet. 2015 Sep 9;11(9):e1005494. doi: 10.1371/journal.pgen.1005494 (PMC4564284; doi:10.1371/journal.pgen.1005494)
Supplement: S4 Table — Only polymorphisms within the coding region are represented here. * indicates an amino acid change as a result of the SNP. SNPs #1 and #2 were found in following accessions; TueV13, TueWa1-2, TueScha9, ICE173, ICE191, ICE102, Mer-6, Ped-0, ICE50, ICE49, Vash-1, Lag2.2 ICE63, Kastel-1, ICE138. SNP #3 was found in following accessions; ICE72, ICE61, ICE60, Yeg-1, ICE29. SNP #4 was identified in following accessions: Nie1-2, ICE216, ICE212, ICE213, ICE119, ICE112, Bak-2. (PDF) [file pgen.1005494.s009.pdf]

SNP number	CENH3 Region	AT1G0137 0.1 Coding region base number	Base in AT1G0137 0.1	SNP identified	Coded in AT1G0137 0.1	New codon	Amino acid in AT1G0137 0.1	New amino acid
#1	Tail	60	C	T	GCC	GCT	Alanine	Alanine
#2	Tail	62	G	C	GGT	GCT	Glycine	Alanine *
#3	Tail	150	T	A	CCT	CCA	Proline	Proline
#4	HFD	495	T	C	TTT	TTC	Phenylalanine	Phenylalanine
