# Supplementary material for: Point Mutations in Centromeric Histone Induce Post-zygotic Incompatibility and Uniparental Inheritance
Source: PLoS Genet. 2015 Sep 9;11(9):e1005494. doi: 10.1371/journal.pgen.1005494 (PMC4564284; doi:10.1371/journal.pgen.1005494)
Supplement: S5 Table — Column 4 indicates the amino acid changes tested in this study and column 5 indicate if that particular mutation can act as a haploid inducer (highlighted in green) or not (highlighted in magenta). (PDF) [file pgen.1005494.s010.pdf]

Amino acid (AA) position in *A.thaliana*	Amino acid	Conserved/Total	AA change in transgenic lines	Haploid induction
82	P	42/53	P82S	YES
83	G	53/53	G83E	YES
84	T	50/53		
85	V	44/53		
86	A	53/53	A86V	YES
87	L	52/53		
88	K	2/53		
89	E	53/53		
90	I	53/53		
91	R	53/53		
92	H	17/53		
93	F	36/53		
94	Q	53/53		
95	K	50/53		
96	Q	4/53		
97	T	25/53		
98	N	22/53		
99	L	47/53		
100	L	50/53		
101	I	52/53		
102	P	53/53	P102S	NO
103	A	37/53		
104	A	51/53		
105	S	17/53		
106	F	53/53		
107	I	36/53		
108	R	53/53		
109	E	9/53		
110	V	51/53		
111	R	35/53		
112	S	16/53		
113	I	46/53		
114	T	36/53		
115	H	16/53		
116	M	2/53		
117	L	11/53		
118	A	33/53		
119	P	15/53		
120	P	34/53		
121	Q	13/53		

122	I	22/53		
123	N	9/53		
124	R	53/53		
125	W	50/53		
126	T	38/53		
127	A	36/53		
128	E	51/53		
129	A	53/53		
130	L	46/53		
131	V	27/53		
132	A	53/53	A163T	YES (Weak)
133	L	42/53		
134	Q	53/53		
135	E	53/53		
136	A	53/53	A136T HI	YES
137	A	52/53		
138	E	53/53		
139	D	36/53		
140	Y	22/53		
141	L	48/53		
142	V	44/53		
143	G	16/53		
144	L	43/53		
145	F	51/53		
146	S	16/53		
147	D	38/53		
148	S	28/53		
149	M	37/53		
150	L	51/53		
151	C	53/53		
152	A	51/53		
153	I	53/53		
154	H	53/53		
155	A	53/53		
156	R	14/53		
157	R	53/53		
158	V	49/53		
159	T	53/53		
160	L	39/53		
161	M	53/53		
162	R	16/53		
163	K	53/53		
164	D	53/53		

165	F	28/53		
166	E	36/53		
167	L	53/53		
168	A	53/53		
169	R	53/53		
170	R	53/53		
171	L	34/53		
172	G	48/53		
173	G	49/53	G173E	NO
174	K	34/53		
175	G	34/53		
176	R	40/53		
177	P	46/53		
178	W	47/53		
