# Supplementary material for: Point Mutations in Centromeric Histone Induce Post-zygotic Incompatibility and Uniparental Inheritance
Source: PLoS Genet. 2015 Sep 9;11(9):e1005494. doi: 10.1371/journal.pgen.1005494 (PMC4564284; doi:10.1371/journal.pgen.1005494)
Supplement: S6 Table — (A) Variant amino acid residues from different species at position corresponding to residue 82 from A. thaliana. (B) Variant amino acid residue from different species at position corresponding to residue 173 from of A. thaliana. Highlighted in yellow (A) are the species that carry P82S polymorphisms. O. alta and O. minuta are alloteraploid rice species that carry CCDD and BBCC genomes respectively. (PDF) [file pgen.1005494.s011.pdf]

A

Species	Amino acid at position 82 in *A. thaliana*	Amino acid at the corresponding position in this species
Actinidia chinensis	P	S
Brachypodium distachyon	P	A
Crucifera himalaica	P	A
Glycine max	P	S
Gossypium hirsutum	P	A
Oryza alta (C genome)	P	S
Oryza minuta (C genome)	P	S
Oryza rhizomatis	P	S
Populus trichocarpa	P	S
Saccharum officinalis	P	V
Sorghum bicolor	P	A

B

Species	Amino acid at position 173 in *A. thaliana*	Amino acid at the corresponding position in this species
Carthamus tinctorius	G	K
Cichorium intybus	G	K
Helianthus exilis	G	K
Lactuta sativa	G	K
